# Artificial light at night bans *Chaoborus* from vital epilimnetic waters

**DOI:** 10.1038/s41598-024-58406-y

**Published:** 2024-04-05

**Authors:** Mirosław Ślusarczyk, Anna Bednarska, Marcin Lukasz Zebrowski, Joanna Tałanda

**Affiliations:** 1 Department of Hydrobiology, Institute of Functional Biology and Ecology, Faculty of Biology, Biological and Chemical Research Centre, University of Warsaw, Żwirki I Wigury 101, 02-089 Warsaw, Poland; 2 Hydrobiological Station, Faculty of Biology, University of Warsaw, Pilchy 5, 12-200 Pisz, Poland

**Keywords:** Ecology, Physiology

## Abstract

Artificial light at night (ALAN) is known to affect organisms in terrestrial ecosystems and adjacent litoral habitats. In the present study, we tested the effect of ALAN on the spatial distribution of organisms in open waters, using the insect larvae of *Chaoborus flavicans* as an example. During the day *C. flavicans* typically hide from visually hunting fish in deep, dark, anoxic waters. On safer nights, they forage in rich subsurface waters. Nighttime field tests revealed that light from an HPS street lamp mounted on a boat anchored in open water attracted planktivorous fish, but deterred planktonic *Chaoborus* from rich but risky surface waters. *Chaoborus* did not descend to the safest, anoxic hypolimnion, but remained in hypoxic mid-depth metalimnion, which does not appear to be a perfect refuge. Neither light gradient nor food distribution fully explained their mid-depth residence under ALAN conditions. A further laboratory test revealed a limited tolerance of *C. flavicans* to anoxia. Half of the test larvae died after 38 h at 9 °C in anoxic conditions. The trade-off between predation risk and oxygen demand may explain why *Chaoborus* did not hide in deep anoxic waters, but remained in the riskier metalimnion with residual oxygen under ALAN conditions.

## Introduction

Artificial Light at Night (ALAN) is an increasing global scale phenomenon that alters day/night light regimes and leads to environmental light pollution^1^. Highly populated settlements (which are the main source of ALAN), are often located on waterfronts^2^. Thus ALAN may also affect littoral zone of seas^3^, lakes and rivers^4–6^. Studies focusing on the effects of ALAN on aquatic organisms in open waters are scarce, despite its potential significance^7,8^.

Aquatic organisms are sensitive to natural vertical light gradients. In aquatic ecosystems, sunlight (the primary source of light energy) intensity decreases exponentially with water depth due to the strong absorption and reflection of light by water and soluble and suspended matter^9^. In stagnant waters during periods of intense solar activity, the heterogeneous distribution of light energy leads to a vertical thermal stratification with warm, low-density water near the surface (epilimnion), cold, denser water above the bottom (hypolimnion), and an intermediate layer with a steep vertical temperature gradient in between (metalimnion). This thermally driven vertical gradient in water density and viscosity creates an invisible barrier to water circulation in the metalimnion, significantly reducing the exchange of oxygen (and other dissolved substances) between the well oxygenated, illuminated epilimnion and the dark hypolimnion, which can become anoxic in waters with high oxic demand in most mesotrophic and eutrophic lakes^9^.

Moreover, sunlight may be an important source of information about current or future environmental conditions, including the distribution of food and the predation risk^10^. Both prey and predators may use direct or indirect light cues and modify their activity and distribution to minimize risks and maximize gains of trophic interaction in spatially and temporarily heterogenous waters^11,12^. Fish are considered keystone predators in many aquatic habitats^13^. They may forage in darkness, yet light improves their hunting efficiency^14^. On the other hand, light may expose fish to their own predators like piscivorous fish and birds. In lakes, the foraging by planktivorous fish may not be the most intense during the day, when light conditions are most favorable, but at dusk and dawn, when they are relatively safe from their own predators, but their visual foraging is still possible during the period of so-called antipredation time window^15^. When exposed to visual fish predation, their potential prey may avoid illuminated subsurface waters by spending daylight hours in deeper, darker, albeit cold and resource-poor waters, and returning to more favorable surface waters at night, when fish foraging efficiency is low. This phenomenon is known as Diel Vertical Migration (DVM) behavior and is exhibited by a wide range of fish prey belonging to wide range of zooplankton taxa^16,17^. While the phenomenon of diurnal migration is reproducible throughout the day/night cycle, it can sometimes, be disrupted by reflected moonlight during a full moon in cloudless weather, resulting in strong predation pressure even at night^18^.

One of the most sensitive aquatic animals to cyclical changes in light intensity are large planktonic invertebrates, including insect larvae of *Chaoborus*, the subject of the present study. *Chaoborus* larvae are key invertebrate predators in the pelagic zone of many lakes and ponds^19,20^. It is a bi-environmental insect with four larval and one pupal stages living in aquatic environments and a short-lived adult stage that mates in aerial habitats. The larvae are tactile, voracious predators feeding on planktonic cladocerans, copepods and rotifers^19–22^. In turn they are readily eaten by planktivorous fish due to their relatively large size compared to other planktonic animals^23^. In shallow or transparent, well oxygenated oligotrophic and mesotrophic lakes, where fish can forage throughout the water column, *Chaoborus* larvae avoid fish during the day by burrowing into the soft sediments^24^. In eutrophic lakes, however, *Chaoborus* usually do not burrow into the sediment, but instead use the dark, anoxic, cold hypolimnion as a daytime refuge from fish when the lack of oxygen in the hypolimnion in summer deters fish from foraging in the deep water^25^. Both strategies employed by *Chaoborus* require periodic tolerance to anoxia. *Chaoborus* larvae are known for temporal tolerance to oxygen deficits lasting hours according to some sources^26^ or weeks according to others^27,28^. At dusk, as the sunlight fades, they move up into the warm, oxygenated and food-rich epilimnetic waters where they spend the short summer nights. They return to the deep waters at dawn^29^. Existing data suggest that the cyclic nocturnal migration of the closely related *C. crystallinus* and *C. punctipennis* from deep anoxic to normoxic surface waters is driven by the need to inactivate the toxic residual metabolites accumulated during daily anoxic metabolism and “repay the oxygen debt”^26,30^. The ultimate function of DVM in *Chaoborus* appears to be a trade-off between avoidance of fish predation by remaining in safer, darker, anoxic deeper waters during risky daytime hours and the demand for resources (food and oxygen) and favorable environmental conditions (elevated temperature) safely acquired at night in subsurface waters^24,31^. Residing permanently in deep, cold, anoxic water depleted of resources would be maladaptive, if at all possible.

The relatively new anthropogenic disturbance such as artificial light at night can be expected to affect the natural distribution of organisms in the water column and disrupt the diurnal vertical migrations of various aquatic organisms as the DVM are essentially driven by light conditions^32–34^. ALAN is mostly caused by highly populated, urbanized areas which illuminate surrounding terrestrial ecosystems and adjacent litoral zones of aquatic habitats^32,35^. However, in some locations, ALAN may also have the potential to affect the pelagic zone as well under illuminated bridges, boats, cruise ships or floating platforms, within expanding wind farms and in the vicinity of intensely lit settlements (urban lights reflected by clouds)^33,36^. Freshwater bodies have been rarely studied for the presence and intensity of ALAN^5^. There is a lack of satellite data, and in-situ measurements vary depending on the type and sensitivity of the instruments or the unit of measurement^5,32^. Nevertheless, the maximum intensity of artificial light at night may exceed the intensity of moonlight during full moon (0.05–0.3 lx) but it is orders of magnitude lower than the potential intensity of sunlight (130,000 lx)^32^.

To date, there are relatively few studies analyzing the possible effects of ALAN on aquatic organisms in open water. It was shown that ALAN may attract pelagic fish^36–38^, enhance their foraging activity^39–41^ and increase their body mass through longer foraging time and prey attraction^42,43^. In turn, some planktonic animals may respond to artificial light by reducing the range of DVM^7^, which may inhibit their growth rate in thermally stratified waters^44^. On the other hand, ALAN may create a trap for some planktonic animals by attracting them to the light source^42,45,46^.

In the present study, we aimed to experimentally investigate the potential effect of ALAN on the nocturnal distribution of intensively migrating *C. flavicans* larvae in an offshore zone of a dimictic eutrophic lake with a strong vertical summer temperature and oxygen gradient. We expected artificial light to have a strong deterrent effect on its vertical distribution at night. In addition, we aimed to verify conflicting information on the scope of temporal tolerance of *C. flavicans* larvae to anoxic conditions and to assess its potential effect on their DVM pattern when exposed to ALAN.

## Results

### Environmental conditions

**Figure 1:**
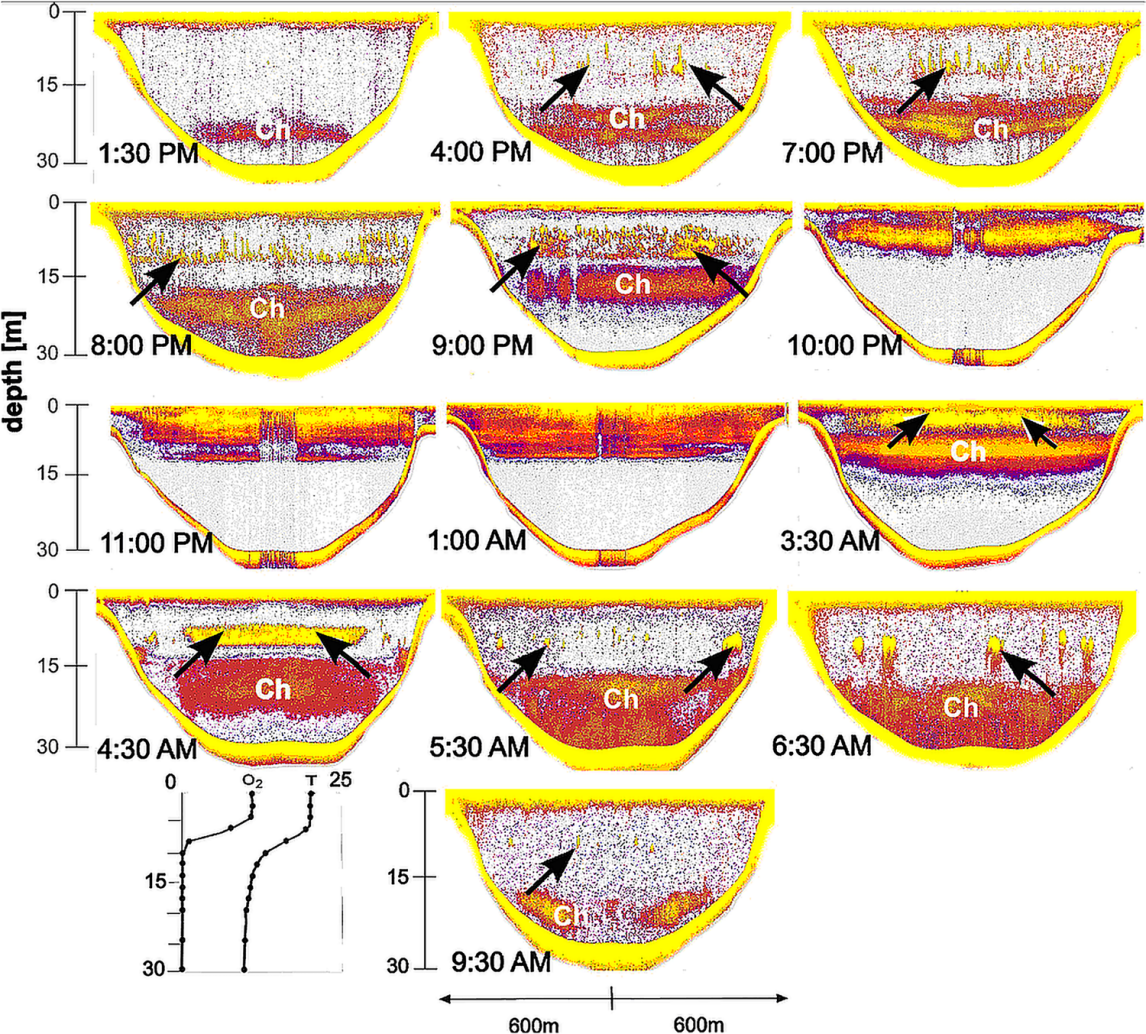
The natural diurnal pattern of spatial distribution of *C. flavicans* larvae and fish in the main basin of Lake Roś in July 2015. White color indicate water, while warm colors indicate sound reflecting objects in the water. Black arrows indicate the presence of fish, “Ch” indicates the presence of *Chaoborus* larvae. A single transect extending from the littoral zone to the deepest point and back. Vertical gradients of temperature (measured in °C) and oxygen (measured in mg × L^−1^) along the water depth in the lake are shown in the lower left corner.

**Figure 2:**
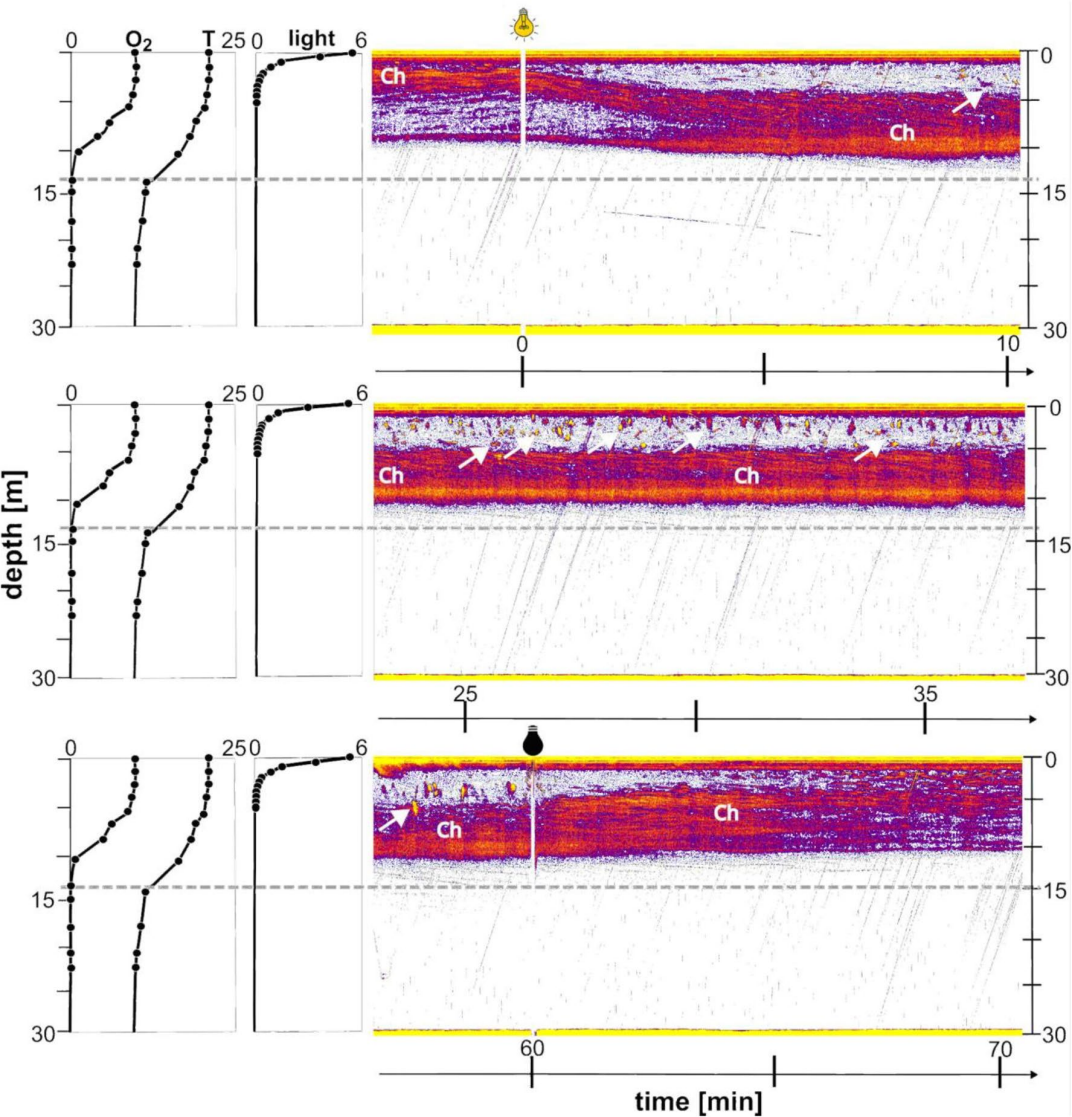
Example of temporal dynamics of vertical distribution *of Chaoborus flavicans* and fish under the lamp before, during and after artificial illumination of the pelagic zone in Lake Roś (on the right—“Ch”—presence of *Chaoborus*; white arrows point to fish). The grey dashed line represents the beginning of the anoxic layer. Left panel shows the vertical distribution of artificial light and physicochemical parameters during the ALAN phase in Lake Roś (O_2_: oxygen concentration (mg × L^−1^); T: temperature (°C); Light: photon flux (μmol × m^−2^ × s^−1^). Photon flux corresponds to ALAN treatment only.

Biotic conditions, most favorable for aerobic organisms, was observed in the warm (20.5 ± 0.5 °C), well oxygenated (9.2 ± 0.5 mg × L^−1^) epilimnion that ranged from the surface to 6 m depth (Figs. 1, 2). The metalimnion extended from 6 to 15 m with steep temperature drop from 20.5 to 11.5 °C respectively. Oxygen content dropped from 8.8 mg L^−1^ at 6 m, through 3.5 mg × L^−1^ at 10 m; and 0.7 mg × L^−1^ at 10.5 m, to 0.0 mg × L^−1^ at 13 m and below. Hypolimnetic anoxic waters with a low temperature of 10.5 ± 1 °C extended to the bottom of the lake.

### Natural distribution of the *Chaoborus* and fish

The diurnal pattern of the spatial distribution of the *Chaoborus* larvae and fish recorded by sonar in the lake Roś in 2015 (Fig. 1) is a typical distribution of those animals in eutrophic lakes with anoxic hypolimnion in summer . At midday most *Chaoborus* resided deep (20–25 m) in the water column a few meters above the bottom in cold, dark anoxic waters. Fish were not visible on the echogram in that strata, at that time. Fish in shoals were visible in the epilimnetic waters in the further daytime hours. At dusk, pelagic shoals of fish dispersed and density of fish in the epilimnetic waters increased most likely due to horizontal migration from the littoral, while *Chaoborus* larvae rose toward the surface. At 8:00 p.m. and 9:00 p.m. *Chaoborus* larvae and fish were still separated vertically, but later on, as light intensity further decreased, they started to share the epilimnetic zone, the co-occurrence in the surface water lasted from 10:00 p.m. until dawn. Strong acoustic signals reflected from the dense *Chaoborus* population overshadowed the fish signals on the echograms, so it is difficult to determine fish distribution at that time. At dawn (3:30 a.m.) *Chaoborus* larvae resided lower than fish in the epilimnion. Then it descended to hypolimnetic waters when the light intensity increased. Fish moved deeper in the epilimnion, grouped together in schools or likely migrated toward the littoral zone in fear of predation by piscivorous fish or birds. At 9:30 a.m. *Chaoborus* larvea remained in the deep hypolimnion and its vertical distribution circled back to resemble the situation at 1:30 p.m.

### ALAN effect

#### Plankton samples analysis

**Figure 3:**
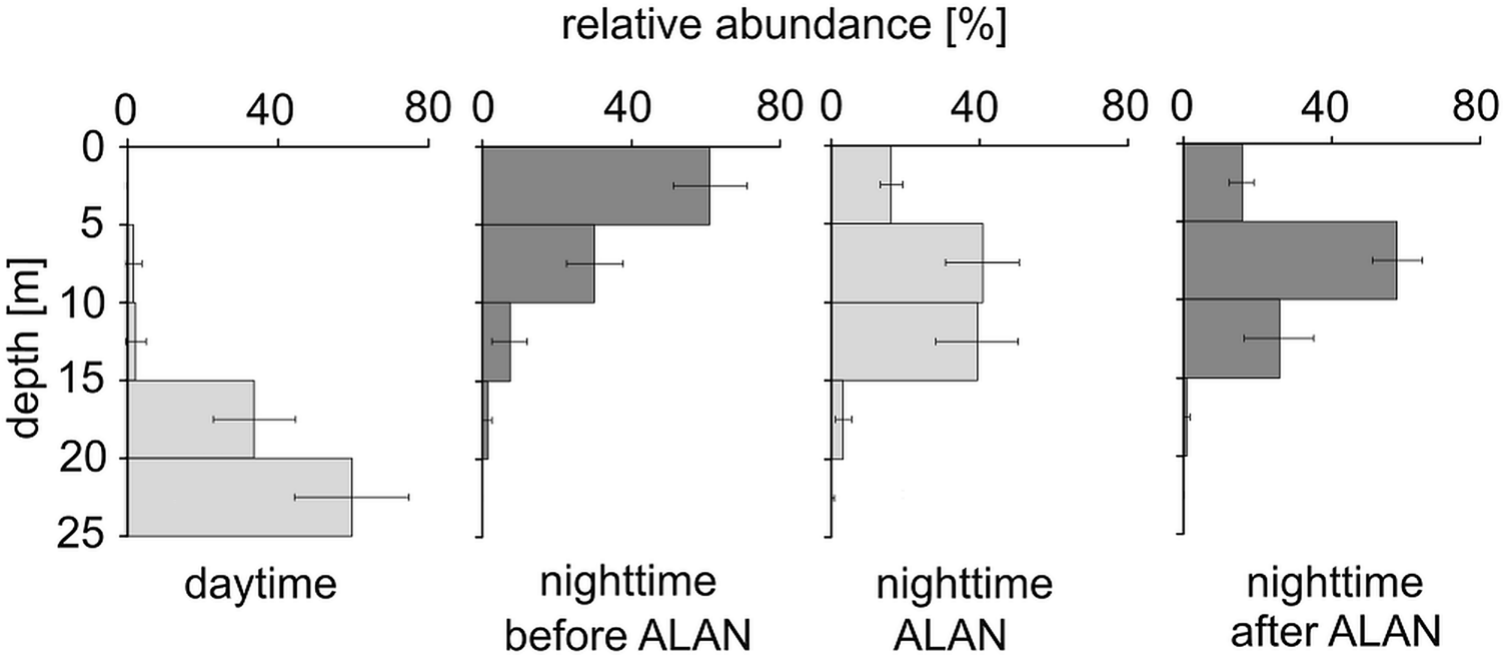
Vertical distribution of *Chaoborus* larvae (mean proportion ± 1SE) during the day, at night before ALAN, at night during ALAN and at night after ALAN.

Analysis of the zooplankton samples revealed that the majority of *Chaoborus* individuals were in the IVth larval stage of development during our study in the lake with larval density reaching 703.6 ± 87.2 ind. × m^−2^ and 28.1 ± 3.5 ind. × m^−3^ (mean ± 1SE). Statistical analysis of zooplankton densities showed that the total abundance of *Chaboborus* larvae did not differ between the light conditions (Table 1), while it varied significantly between depths (Table 1; Table 1 in the Supplementary Material). The interactions between the two factors were significant (Table 1), indicating that light conditions affected *Chaoborus* abundance differently at different depths. During the day, *Chaoborus* density was significantly higher in the deep, dark and anoxic layers of the lake (15–20 and 20–25 m) compared to the upper layers (0–5, 5–10 and 10–15 m) (Fig. 3; Table 2 in the Supplementary Material). At night, before exposure to ALAN, we observed an inverse distribution of *Chaoborus* in the lake compared to the daytime period. *Chaoborus* abundance was significantly higher in upper, warm and oxygenated layers (0–5, 5-10 m) than in lower lake layers (10–15, 15–20 and 20–25 m) (Fig. 3; Table 2 and 3 in the Supplementary Material). During ALAN exposure *Chaoborus* abundance was the highest in the middle water layers (5–10 and 10–15 m), slightly lower in the surface layer (0-5 m), and lowest in the deepest layers (15–20, 20–25 m) (Fig. 3; Table 2 in the Supplementary Material). *Chaoborus* abundance during ALAN exposure was significantly higher in the upper layers (0–5, 5–10, 10–15 m) and lower in the deepest layers (15–20, 20–25 m) compared to daytime (Fig. 3; Table 3 in the Supplementary Material), while it was significantly lower in the 0–5 m surface layer and much higher in the 10–15 m middle layer compared to nighttime (Fig. 3; Table 3 in the Supplementary Material). After exposure to ALAN, the highest abundance of *Chaoborus* was found in the 5–10 m layer, slightly lower but significantly different in the 0–5 and 10–15 m layers, while *Chaoborus* was virtually absent in the deepest layers (15–20, 20–25 m) devoid of oxygen (Fig. 3; Table 2 in the Supplementary Material). However, we did not observe any significant differences in *Chaoborus* abundance for any depth between ALAN and darkness 1 h after turning off the light (Fig. 3; Table 3 in the Supplementary Material).

**Table 1:** Table of the deviance analysis of the generalized linear mixed effects model to test the effect of depth and treatments and their interaction on the abundance of *Chaoborus* larvae in the lake.

Factors; interaction	χ^2^	df	*p*
Treatment	0.597	3	0.897
Depth	**28.322**	**4**	** < 0.001**
Treatment × depth	**220.097**	**12**	** < 0.001**

Statistically significant differences (α = 0.05) are shown in bold.

χ^2^, Chi-squared test; df, Degrees of freedom, *p*, *p* value.

**Figure 4:**
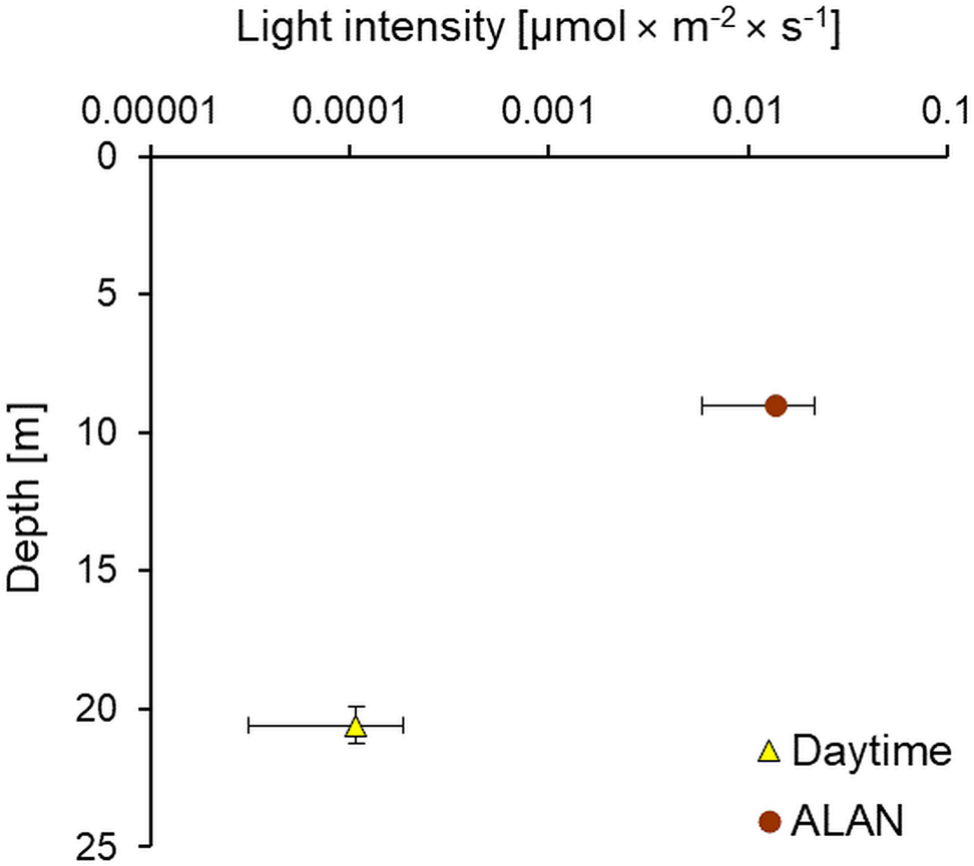
Mean light intensity (± 1SE) at mean (± 1SE) depth of the *Chaoborus* population during daytime compared to ALAN conditions in Lake Roś.

During the day, light intensity at the mean depth (20.1 ± 1.2 m) of the *Chaoborus* population occurrence was estimated (extrapolated) to be 1.1 × 10^−4^ ± 0.8 × 10^−4^ μmol × m^−2^ × s^−1^ (Fig. 4). ALAN deterred *Chaoborus* from the uppermost zone (0–5 m) to deeper waters at a mean depth of 9.0 ± 0.4 m, where the estimated light intensity was 1.4 × 10^−2^ ± 0.8 × 10^−2^ μmol × m^−2^ × s^−1^ (Fig. 4). The light intensity for the average depth of the *Chaoborus* population under ALAN conditions was thus two orders of magnitude higher (with marginal significance according to the Wilcoxon rank sum test, W_1, 9_ = 22, p = 0.056) and demonstrated a two orders of magnitude higher variability of this parameter (Fig. 4).

ALAN did not seem to affect the vertical distribution of pelagic crustaceans—potential *Chaoborus* prey (Fig. 1 in the Supplementary Material).

#### Sonar recordings analysis

**Figure 5:**
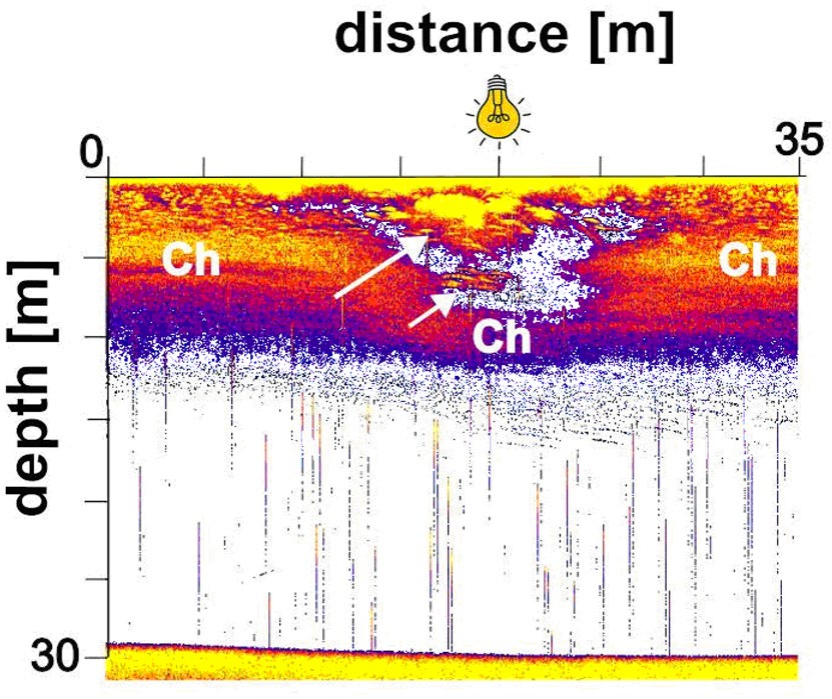
Cross-sectional echogram recorded 30 min after the light was turned on. Light bulb indicates the location of an artificial light source. White arrows indicate the presence of fish, “Ch” indicates the presence of *Chaoborus* larvae.

The sonar recordings made it possible to track the distribution of *Chaoborus* larvae in more detail. The sonar graph indicates that at midnight most larvae were in the warm, oxygenated epilimnion between 0 and 6 m, with few below (Fig. 2). Minutes after the light was switched on, we could observe below the lamp fast descending of the larvae along with an increasing number of fish appearing above them (Fig. 2). Ten minutes after the light phase began, *Chaoborus* larvae could hardly be detected on the echograms between 0 and 5 m, with some of them observed between 5 and 9 m and most of them between 9 and 13 m in the hypoxic zone. Few individuals were visible below 13 m in the anoxic zone at that time. Over time of illumination the density of fish increased and they began to form shoals in subsurface water. The cross sectional echogram taken by the cruising boat 2 m away from the lamp during the ALAN phase revealed a relatively narrow range of spatial effect of the applied light on the *Chaoborus* distribution. The artificial light affected distribution of *Chaoborus* larvae within 12 m radius from the lamp approximately (Fig. 5). With the light off, the schools of fish dispersed almost immediately and their density decreased below the lamp as they lost the attractant point. At the same time, *Chaoborus* gradually began to rise to the surface (Fig. 2). The upward movement of *Chaoborus* after the light was turned off seemed slower than the downward one after the light was switched on (Fig. 2).

#### *C. flavicans* larvae tolerance to anoxic conditions

**Figure 6:**
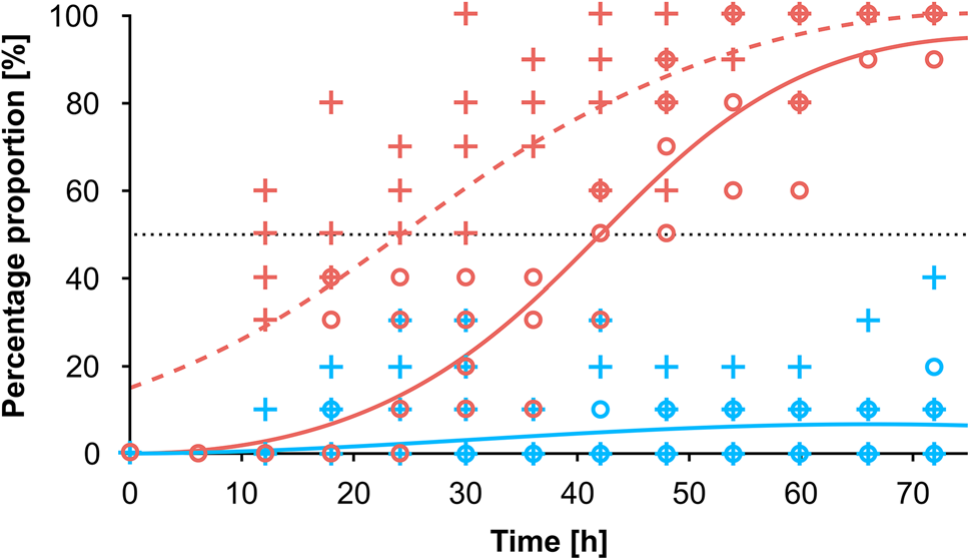
Effect of time on mortality of *Chaoborus* larvae in anoxic (open red circles) and normoxic (open blue circles) conditions with logistic regressions (solid lines in corresponding color) fitted to the experimental data. Prevalence of experimental animals at the water surface over time is shown with plus marks in anoxic (red) and normoxic (blue) conditions. The dashed line shows the logistic regression fitted to the data on surface appearance in anoxic conditions. No logistic regression could be fitted to the surface appearance data in the normoxic conditions.

Survival analysis revealed that survivorship of *C. flavicans* larvae in the anoxic conditions was lower compared to normoxic ones (Fig. 6; Fig. 2 in the Supplementary Material). Only 4% of all tested larvae stayed alive under 72 h of anoxic conditions compared to 92% in the normoxic ones at the temperature of 9 °C (Fig. 6). The best-fit logistic regression model for the data on mortality of *Chaoborus* larvae (M) as a function of time in anoxia was empirically estimated as M = 0.98375/(1 + 40.86260 × exp(− 0.09741 × T)), where M is the mortality rate and T is the time in hours in anoxia. According to the logistic regression model, the LT50 of *Chaoborus* larvae in anoxia (indicating the time of anoxia that causes 50% mortality in experimental animals) was 38 h 25 min.

A few hours before physiological death the larvae used to appear at the water surface in the experimental chambers (Fig. 6). Survival analysis indicated that the prevalence of *Chaoborus* larvae on the water surface over time differed statistically between normoxic and anoxic conditions (Fig. 6; Fig. 3 in the Supplementary Material). The hazard ratio expressing effect size was significantly higher under anoxia than normoxia assumed as the reference (Fig. 6; Fig. 3 in the Supplementary Material). Half of the tested population appeared at the water surface in experimental chambers after 22 h and 9 min of anoxic conditions. The best-fit logistic regression model for the data on their surface occurrence (S) as a function of time in anoxia was empirically estimated as S = 1.04029/(1 + 25.12443 × exp(− 0.14216 × T)) where S is the surface prevalence rate and T is the time in anoxia in hours.

## Discussion

Our study evidenced a strong effect of artificial light on the distribution of fish and *Chaoborus* in the middle of a lake at night. Within seconds of turning on the light, young fish began to appear in the vicinity of the lamp, and their density increased rapidly (Fig. 2). Within minutes, the incoming fish began to school, as they typically do during daylight hours (Fig. 1), probably as a defense mechanism against piscivorous fish that may have also been attracted by the artificial light^37^. A number of fish dispersed into the deeper and darker epilimnetic waters (Figs. 2, 5), where they probably continued to feed on zooplankton. Unfortunately, the determination of fish identity, density and their foraging pressure was not within the scope of our study. Our results are consistent with previously published findings on the effects of ALAN on fish behavior in pelagic waters^36,37^.

A novel finding of our study is the strong effect of ALAN on the distribution of *Chaoborus*. When exposed to artificial light, unlike fish, *Chaoborus* larvae rapidly descended from the resource-rich epilimnion to the cooler, hypoxic metalimnion and remained there until the light was turned off (Figs. 2, 3), probably as a defense against the increased risk of fish predation. Downwards movements of *Chaoborus* larvae were considerably faster than upward movements after the light was turned off (Fig. 2), which could be interpreted as a cautious response to anticipated surface risk. *Chaoborus* larvae descended a few meters into poorly lit, hypoxic metalimnetic waters presumably in response to the risk of fish predation increased by ALAN. Interestingly, only a small fraction of larvae were observed below 13 m (below the oxycline but still in the metalimnion) under ALAN conditions (Figs. 2, 3), while most of them were found much deeper during the daytime hours (Figs. 1, 3). Why did most of the *Chaoborus* not migrate deeper into the safer hypolimnetic water under artificial light, where fish rarely venture? Could they feel safe in metalimnetic waters due to low light intensity under ALAN conditions or low oxygen levels which should reduce fish predation pressure? It seems unlikely. First the larvae under ALAN conditions resided in metalimnetic water at two orders of magnitude higher light intensity (1.4 × 10^−2^ ± 0.8 × 10^−2^ μmol × m^−2^ × s^−1^ at mean population depth) compared to their daytime residence depth in anoxic hypolimnetic refugia (1.1 × 10^−4^ ± 0.8 × 10^−4^ μmol × m^−2^ × s^−1^ at mean population depth). In addition, *Chaoborus* experienced/'tolerated' two orders of magnitude greater variability in light conditions under artificial light at night than during daylight hours. Finally, fish are able to temporarily dive into oxygen-deficient waters in complete darkness in search of food^47,48^. Therefore, *Chaoborus* should not have felt safe in metalimnion under ALAN conditions. What could prevent *Chaoborus* from hiding in the deeper and safer strata under ALAN conditions? Risky residence in metalimnetic waters is unlikely to be driven by the need to reduce the energetic costs associated with long-range vertical movement, as these costs appear to be marginal due to the adoption of a hydrostatic mechanism of vertical migration^49^. These might include vertical gradients in temperature, food concentration or oxygen level that are negatively correlated with depth. The slower metabolic rate caused by prolonged residence in deeper, colder waters might not allow *Chaoborus* to complete its life cycle in a given season, under ALAN conditions. The vertical distribution of food could also influence larvae to restrict their descent into the deeper layers of a lake. *Chaoborus* larvae are known to forage near the water surface during dark nights^50,51^. However, further studies are needed to verify the significance of this hypothesis.

The vertical distribution of food could also force larvae to limit their descent into the deep layers of a lake. *Chaoborus* larvae are known to forage near the water surface at night^49^ in complete darkness^51^. Staying deeper in the water column during the night triggered by artificial illumination may have had an impact on its foraging success not because of lower capture efficiency in the dark, but because of the fewer prey it encountered in deeper waters (Fig. 1 in the Supplementary Material). The avoidance of anoxic layers at night seems, however, to be more important than other reasons for the metalimnetic residence of *Chaoborus* under ALAN conditions. Some existing data showed that *Chaoborus* larvae can tolerate anoxic conditions for hours^26,30^ rather than days or weeks suggested by others^27,28^. The cyclic nocturnal migration of the closely related *C. crystallinus* and *C. punctipennis* from deep anoxic to normoxic surface waters has been suggested to be driven by the need to inactivate toxic residual metabolites accumulated during daily anoxic metabolism and to “repay the oxygen debt”^26,30^. In order to test if similar constraints may limit the range of vertical migration of *C. flavicans* in our study, we conducted a laboratory assay that revealed limited tolerance of experimental larvae to anoxia. The data show that, the majority of the most hypoxia-tolerant stage IVth larvae did not survive over 38 h of anoxic conditions (Fig. 6). These temporal limits may be even shorter if we consider the much earlier appearance of the majority of experimental larvae at the water surface in (short) experimental vessels (after 22 h of anoxia), which could cause their ecological mortality (consumption by fish) under ALAN conditions in the field. All this implies that active *C. flavicans* larvae, like *C. cristalinus*, must visit oxygenated waters each night to 'pay' the oxygen debt. While the temperature and food gradients are likely to be important, the vertical gradient of predation intensity and oxygen distribution appear to be more critical than others. The gradient of risk of death due to predation and anoxia have an immediate, strong, and direct effect on the fitness of *Chaoborus* explaining its metalimnetic residence under ALAN conditions.

Unlike *Chaoborus*, no similar or opposite changes in the vertical distribution of planktonic crustaceans were observed during the analysis of the plankton samples (Fig. 1 in the Supplementary Material). These results are rather surprising, as^7^ observed differences in the vertical distribution of crustacean *Daphnia* at lower artificial light intensities in the field than used in the present study. Potential differences in vertical distribution of crustaceans were either absent or undetectably small in our study due to the low resolution of the plankton sampling technique used.

Due to the relatively low height of the lamp used, resulting in a low angle of incidence and high reflection from the water surface, the spatial effect of the ALAN on *Chaoborus* larvae distribution was not extensive in our tests. The light only affected *Chaoborus* distribution within a 15 m radius of the lamp used (Fig. 5). It is likely that a more powerful lamp, mounted higher above the water level and shining for a longer period, could have a more widespread and profound effect than observed in our study. It is likely that constant exposure to high levels of illumination could intensify fish predation pressure and exterminate *Chaoborus* from light-polluted sites, which in turn could alter the composition of the local community of its prey.

For the above reasons we recommend caution when using ALAN in open water to reduce the anthropogenic impact on aquatic communities. Low power lamps, light range limiters or safe light colors can be considered as countermeasures. The use of red light, which is barely visible to most aquatic organisms^52,53^ but visible to humans, may be an option.

## Methods

### Study site

The field study was carried out, at different periods of the day, in the deepest basin (31 m) of lake Roś (Great Masurian Lakes, Poland, Europe; 53.67°N, 21.92°E; Fig. 4 in the Supplementary Material) during summer stratification. Roś is a lowland, postglacial, eutrophic (with 1–2 m transparency of water in summer), dimictic, flow-through lake with an average depth of 8.1 m and an area of 189 ha with a 25% littoral covered mainly with reeds. The pelagic zone of this lake at night in summer was occupied by planktivorous fry of smelt (*Osmerus eperlanus*; L., 1758), perch (*Perca fluviatilis*; L., 1758) and roach (*Rutilus rutilus*; L., 1758) in the mid-eighties of the twentieth century^54^ when the lake was still mesotrophic. Recent published data on pelagic fish composition are not available, but our observations indicate that small perch and bleak (*Alburnus alburnus*; L., 1758) have recently been the most abundant fish in the open water at night (unpublished data).

### Measurements of environmental conditions

Temperature and oxygen concentrations at various depths (Fig. 2) were measured during the tests using a submersible optical dissolved oxygen probe (YSI ProODO). Light intensity at different depths marked on the graphs was measured under the lamp during the experiment at night and day using a portable light meter (LiCor LI-250A) with a spherical underwater PAR quantum sensor (LiCor LI-193R) and on a few occasions for comparison using a commercial portable non-submersible Luxometer (Standard Instruments ST-1308 Light Meter; more details at thecalibrationcentre.co.uk) at the water surface.

### Assessment of diurnal natural distribution of fish and *Chaoborus*

The swim bladders of planktivorous fish and the gas sacs of the phantom midge strongly reflect sound waves in the water, which makes them detectable by sonars^55^. In the present study, a commercial fishing sonar (Lowrance LMS-337c) with a 200 kHz transducer mounted on a cruising boat was used to monitor the spatial distribution of fish and *Chaoborus* larvae over the course of a day in the main basin of the lake along a single transect extending from the littoral zone to the deepest spot and back on 20–22 June 2015 (Fig. 1).

### ALAN effect on vertical distribution of aquatic animals in the pelagic zone

A street lamp with High Pressure Sodium (HPS) bulb of medium power (70 W) (luminaire: FORT MTH-413/70W-B, Kanlux; OSRAM) was used to assess effect of artificial light at night on vertical distribution of pelagic animals. HPS lamps are slowly replacing by LEDs, however in Poland HPS are still widely used. The light spectrum of the lamp is shown on the graph (Fig. 5 in the Supplementary Material). The lamp was mounted on a rail on the side of a boat, relatively low—1.5 m above the water, which “compensated” for its lower power compared to the 250–400 W street lamps more commonly used and mounted at a higher level. The boat with the lamp was double anchored near the deepest spot of the lake far from illuminated settlements (Fig. 4 in the Supplementary Material). The maximum ALAN intensity during the experiments reached 250–450 lx under the lamp at the water surface when measured with the non-submersible luxometer, while 3.3–5.9 µmol × m^−2^ × s^−1^ when measured approximately 3 cm below the water surface with the PAR underwater quantum sensor. While these light levels were higher than that documented along the seacoast by^56^, it can be experienced near brightly lit marinas or floating restaurants (unpublished data). The vertical distribution of phantom midge below the lamp was determined upon direct zooplankton catches and, in addition, using sonar (Lowrance ELITE-Ti with 200 kHz transducer) on five summer nights between 4 and 25 July 2019. At that time, the sun set at 8.30 p.m. and rose at 4.30 a.m. Moonlight periodicity was much more variable in that period. Max moon light intensity (0.3 lx)^57,58^ is 3–4 orders of magnitude lower than the artificial light we used in our tests, so it should have a rather marginal effect on the results of our study (additional information in the Table 4 in the Supplementary Material). Night work was carried out using dim red headlamps, barely visible by aquatic animals^52,53^. At midnight, before switching on the light, zooplankton samples were collected at five subsequent water layers (one sample at each layer) in descending order (5–0 m, 10–5 m, 15–10 m, 20–15 m, 25–20 m) using a quantitative closing plankton net with an opening diameter of 28 cm and a mesh size of 150 µm. Shortly afterwards, an echo sounder was switched on to provide a continuous record of sound reflecting aquatic animals (*Chaoborus* larvae and fish). After 10 min of echo sounding in natural darkness, the lamp was turned on for an hour and echo sounding continued. At the end of the light period, depth-stratified zooplankton samples were collected in a similar way as before. In three of five experimental nights echo sounding was continued for 1 hour after the light was switched off and stratified zooplankton samples were collected again in the dark. Moreover, at five occasions midday depth-stratified zooplankton samples as well as light intensity were collected in a similar way to determine daytime *Chaoborus* larvae vertical distribution in the lake upon direct catches during the tests. Zooplankton samples were analyzed under stereomicroscope and planktonic crustaceans were assigned to the lowest possible taxonomic level. The insect larvae (IVth instar) were classified into the species *C. flavicans* based on a morphological analysis of mandibular teeth and fan bristles, labial blades and anal fan rays^59–61^. The density distribution of *Chaoborus* larvae at different depths, estimated from plankton samples, was then compared with the sonar data and light intensity. The mean depth of the *Chaoborus* population was calculated on the basis of zooplankton samples according to the following formula $\sum {\left( {n_{1 - 5} \times d_{1 - 5} } \right)} /\sum {n_{1 - 5} }$, where n is the number of *Chaoborus* larvae collected in each of five (1–5) subsequent vertical strata 5 m long each one, while d is the mean depth of each of five (1–5) vertical strata. Light intensity at the mean depth of the population was then compared between the day and ALAN treatments. Due to the limited sensitivity (0.01 μmol × m^−2^ × s^−1^) and length (10 m) of the sensor cable of the submersible light meter, light intensity at the mean depth of the population was estimated (in most cases extrapolated) using the light-depth regression from the collected data from the surface layers, which were evaluated individually.

### Laboratory test on temporal survival of anoxic conditions by *Chaoborus flavicans*

**Figure 7:**
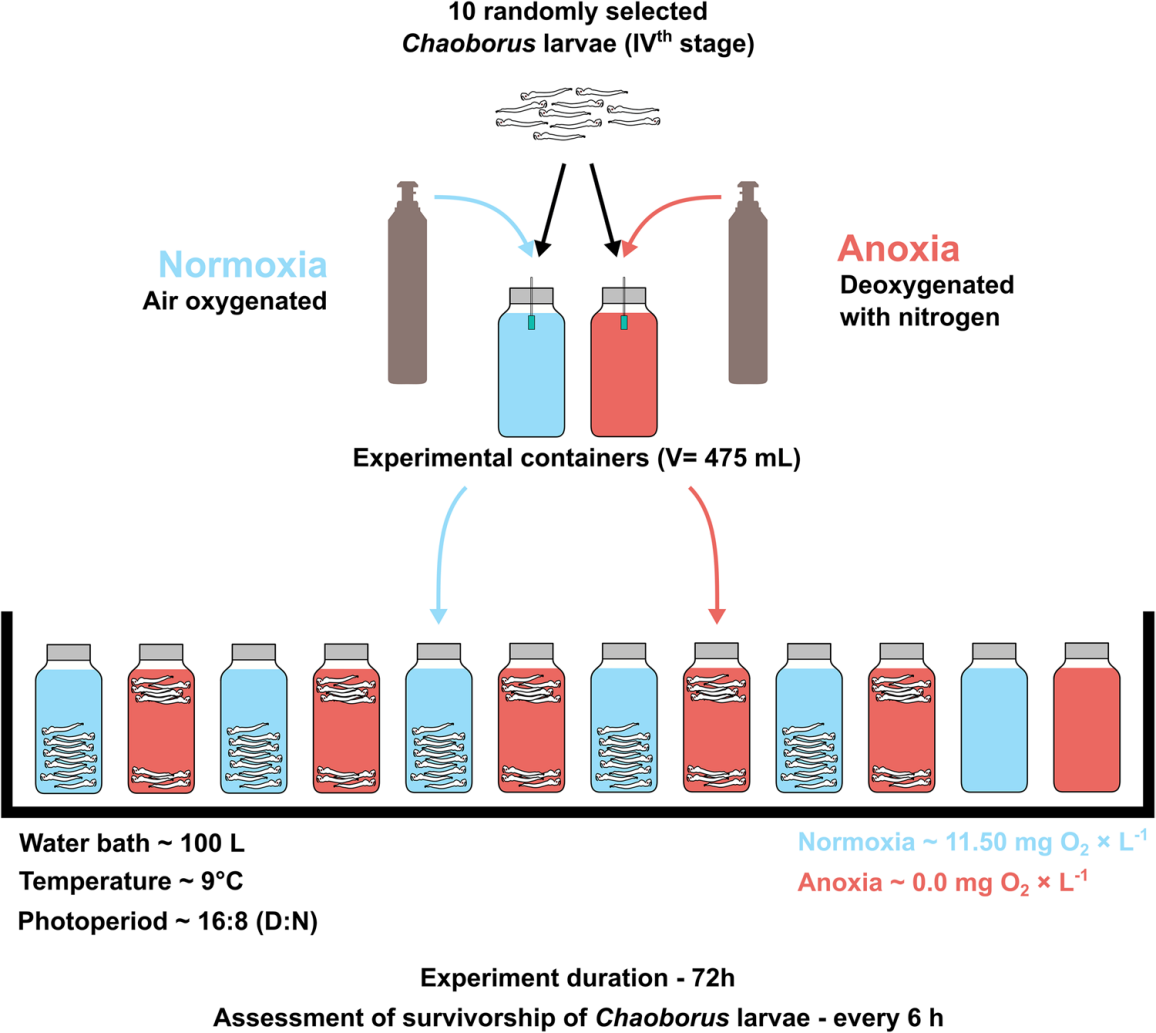
Scheme of the experimental setup on *C. flavicans* larvae tolerance to anoxic conditions.

The experimental animals were collected by towing a plankton net vertically between the depths of 25 and 0 m in the afternoon of July 2022 in Lake Roś. Prior to use in the experiment, the animals were kept in air saturated lake water for about 3 h. 150 *C. flavicans* larvae of similar size in the IVth stage of development were pre-selected from live plankton samples. The experimental setup (Fig. 7) consisted of 12 polyethylene terephthalate (PET) bottles (of 475 mL volume) filled with 450 mL of the lake water without sediment, submerged in the common water bath of controlled temperature, simulating the daytime thermal conditions faced by *Chaoborus* larvae in the hypolimnion of Lake Roś at that time (9 ± 0.5 °C). Each experimental bottle was closed with a tight lid supplied with a gas inlet and outlet. The gas inlet was supplied with a gas diffuser immersed 3 cm below the water surface of the bottle. The gas outlet was located above the water level of the bottle and was equipped with a check valve. Ten randomly selected *Chaoborus* larvae were transferred to each of 10 PET bottles. Two additional bottles without animals were used to periodically measure the oxygen content in the experimental bottles. At the beginning of the experiment, water in six bottles was aerated with an air pump, while in the other six it was vented with nitrogen gas for 3 h. Two hours of water venting with nitrogen proved sufficient to reduce oxygen levels to near-anoxic conditions of about 0.03 mg × L^−1^. Both air and nitrogen ventilation of the water were repeated cyclically for 1 h in the morning and evening of the experimental days. The viability of the experimental animals was assessed every 6 h in each bottle. The criterion for their physiological death was lack of locomotor activity which was typically preceded with surface appearance of barely mobile individuals. The experiment was continued for 72 h, when most of the animals in the anoxic treatment appeared immobile. Experiment complied with the ARRIVE guidelines and was carried out in accordance with the U.K. Animals (Scientific Procedures) Act, 1986 and associated guidelines, EU Directive 2010/63/EU for animal experiments. Due to the lack of clear sex-dependent morphological characteristics, the sex of *Chaoborus* larvae was not determined in either the field study or the laboratory experiment.

### Statistical analysis

Statistical analysis was performed in R v. 4.2.3^62^. For all statistical tests, the significance level was set at α = 0.05. To investigate the effects of treatment and depth and their interaction on the abundance of *Chaoborus* larvae in the lake, we used two-factor generalized linear mixed models (GLMMs^63^) with the template model builder (the glmmTMB package v.1.1.3^64^). The number of *Chaoborus* larvae in the plankton samples was set as the response variable, treatment (daytime, nighttime before ALAN, ALAN, nighttime after ALAN) as the first fixed effect, and depth range (0–5, 5–10, 10–15, 15–20, 20–25 m) as the second fixed effect and random intercept. To better fit the data into the model, a quadratic transformation of the response variable was performed. The count data, after the above transformation, were modeled with a Gaussian regression with a zero-inflated parameter. The significance of the interaction between factors was assessed using the analysis of deviance and the Wald type II Chi-squared difference test (χ^2^) (car package v.3.0-12^65^). The model fit to the data was optimized using the stats:anova() function and visual inspection of the DHARMa scaled residual plots (the DHARMA package v.0.4.5^66^). Post-hoc tests (for both factors and interactions) were performed using planned contrast for estimated marginal means (EMMs; the emmeans package (v.1.7.2^67^). The ‘holm’ *p* value adjustment was applied to control for type I error inflation due to multiple testing.

Light intensity at mean depth of the *Chaoborus* population was compared between the daytime and ALAN conditions using the nonparametric Wilcoxon rank sum test due to unequal variances in the compared data sets.

The effect of time in anoxia on mortality and surface prevalence of laboratory animals was investigated using non-linear least squares (NLS) regression and automatically determined model parameters by using self-starting function (the NLS logistic function in R). The model parameters allowed to estimate LT50—time causing 50% mortality within tested individuals as well as time of appearance of half of the population at the water surface in anoxic conditions. Survival analysis (package “survival” v.3.5-3^68,69^) and the nonparametric Kaplan–Maier method for censored data^70^ with the log-rank (chi square) test were used to compare survivorship of *Chaoborus* larvae between oxygen treatments. The effect size was evaluated using the hazard ratio method and the Cox proportional hazards model^71^.

### Supplementary Information

Supplementary Information.

## Data Availability

The datasets generated during and/or analyzed during the current study are available from the corresponding author on reasonable request.

## References

[1] Longcore T, Rich C (2004). Ecological light pollution. Front. Ecol. Environ..

[2] Hölker F, Jechow A, Schroer S, Tockner K, Gessner MO (2023). Light pollution of freshwater ecosystems: Principles, ecological impacts and remedies. Philos. Trans. R. Soc..

[3] Duarte C (2019). Artificial light pollution at night (ALAN) disrupts the distribution and circadian rhythm of a sandy beach isopod. Environ. Pollut..

[4] Perkin EK (2011). The influence of artificial light on stream and riparian ecosystems: Questions, challenges, and perspectives. Ecosphere.

[5] Jechow A, Hölker F (2019). How dark is a river? Artificial light at night in aquatic systems and the need for comprehensive night-time light measurements. Wiley Interdiscip. Rev. Water.

[6] Sullivan SMP, Hossler K, Meyer LA (2019). Artificial lighting at night alters aquatic-riparian invertebrate food webs. Ecol. Appl..

[7] Moore MV, Pierce SM, Walsh HM, Kvalvik SK, Lim JD (2000). Urban light pollution alters the diel vertical migration of *Daphnia*. Internationale Vereinigung für theoretische und angewandte Limnologie: Verhandlungen.

[8] Tałanda J, Maszczyk P, Babkiewicz E, Rutkowska K, Ślusarczyk M (2022). The short-term effects of planktivorous fish foraging in the presence of artificial light at night on lake zooplankton. J. Plankton. Res..

[9] Wetzel RG (2001). Limnology. Lake and River Ecosystems.

[10] Aksnes DL, Nejstgaard J, Sædberg E, Sørnes T (2004). Optical control of fish and zooplankton populations. Limnol. Oceanogr..

[11] Gliwicz, Z.M. Between hazards of starvation and risk of predation: The ecology of offshore animals. In *Excellence in Ecology*, Vol. 12 (International Ecology Institute, 2003).

[12] Dawidowicz P, Pijanowska J, Wellborn GA, Thiel M (2018). Diel vertical migration of aquatic crustaceans—Adaptive role, underlying mechanisms, and ecosystem consequences. The Natural History of the Crustacea: Life Histories.

[13] Shukla I, Gaynor KM, Worm B, Darimont CT (2023). The diversity of animals identified as keystone species. Ecol. Evol..

[14] Liu Z, Uiblein F (1996). Prey detectability mediates selectivity in a zooplanktivorous cyprinid (*Alburnus alburnus* (L.)). Sitzungsber. Abt..

[15] Clark CW, Levy DA (1988). Diel vertical migrations by juvenile sockeye salmon and the antipredation window. Am. Nat..

[16] Bandara K, Varpe Ø, Wijewardene L, Tverberg V, Eiane K (2021). Two hundred years of zooplankton vertical migration research. Biol. Rev..

[17] Mehner T (2012). Diel vertical migration of freshwater fishes—Proximate triggers, ultimate causes and research perspectives. Freshw. Biol..

[18] Gliwicz ZM (1986). A lunar cycle in zooplankton. Ecology.

[19] Kajak Z, Rybak J (1979). The feeding of *Chaoborus flavicans* Meigen (Diptera, Chaoboridae) and its predation on lake zooplankton. Internationale Revue der gesamten Hydrobiologie und Hydrographie.

[20] Elser MM, Ende CNV, Sorrano P, Carpenter SR (1987). *Chaoborus* populations: Response to food web manipulation and potential effects on zooplankton communities. Can. J. Zool..

[21] Liljendahl-Nurminen A (1996). Prey detectability mediates selectivity in a zooplanktivorous cyprinid (*Alburnus alburnus* (L.)). Sitzungsber. Abt..

[22] Jäger IS, Hölker F, Flöder S, Walz N (2011). Impact of *Chaoborus flavicans*—Predation on the zooplankton in a mesotrophic lake—A three year study. Int. Rev. Hydrobiol..

[23] Galis F, de Jong PW (1988). Optimal foraging and ontogeny; food selection by *Haplochromis piceatu*s. Oecologia.

[24] Voss S, Mumm H (1999). Where to stay by night and day: Size-specific and seasonal differences in horizontal and vertical distribution of *Chaoborus flavicans* larvae. Freshw. Biol..

[25] Weisser M, Hofmann H, Fernández JE, Peeters F (2018). Vertical migration patterns of the different larval instars of *Chaoborus flavicans* and the influence of dissolved oxygen concentrations. Can. J. Fish. Aquat. Sci..

[26] Englisch H, Opalka B, Zebe E (1982). The anaerobic metabolism of the larvae of the midge *Chaoborus crystallinus*. Insect Biochem..

[27] Dawidowicz P (1993). Diel vertical migration in *Chaoborus flavicans*: Population pattern vs individual tracks. Arch. Hydrobiol..

[28] Cole GA (1994). Textbook of Limnology.

[29] Haney JF, Craggy A, Kimball K, Weeks F (1990). Light control of evening vertical migrations by *Chaoborus punctipennis* larvae. Limnol. Oceanogr..

[30] Doubek JP (2018). The effects of hypolimnetic anoxia on the diel vertical migration of freshwater crustacean zooplankton. Ecosphere..

[31] Luecke C (1986). A change in the pattern of vertical migration of *Chaoborus flavicans* after the introduction of trout. J. Plankton Res..

[32] Moore MV, Kohler SJ, Cheers MS, Rich C, Longcore T, Rich C, Longcore T (2006). Artificial light at night in freshwater habitats and its potential ecological effects. Ecological consequences of artificial night lighting.

[33] Berge J (2020). Artificial light during the polar night disrupts Arctic fish and zooplankton behaviour down to 200 m depth. Commun. Biol..

[34] Maszczyk P, Tałanda J, Babkiewicz E, Leniowski K, Urban P (2021). *Daphnia* depth selection in gradients of light intensity from different artificial sources: An evolutionary trap?. Limnol. Oceanogr..

[35] Gaston KJ, Visser ME, Hölker F (2015). The biological impacts of artificial light at night: The research challenge. Phil. Trans. R. Soc. B.

[36] Becker A, Whitfield AK, Cowley PD, Järnegren J, Næsje TF (2013). Potential effects of artificial light associated with anthropogenic infrastructure on the abundance and foraging behaviour of estuary-associated fishes. J. Appl. Ecol..

[37] Ben-Yami M (1988). Attracting Fish with Light.

[38] Nguyen KQ, Winger PD (2019). Artificial light in commercial industrialized fishing applications: A review. Rev. Fish. Sci. Aquac..

[39] Contor CR, Griffith JS (1995). Nocturnal emergence of juvenile rainbow trout from winter concealment relative to light intensity. Hydrobiologia..

[40] Bolton D (2017). Coastal urban lighting has ecological consequences for multiple trophic levels under the sea. Sci. Total Environ..

[41] Czarnecka M, Kakareko T, Jermacz Ł, Pawlak R, Kobak J (2019). Combined effects of nocturnal exposure to artificial light and habitat complexity on fish foraging. Sci. Total Environ..

[42] Mamcarz A, Nowak M (1987). New version of an illuminated cage for coregonid rearing. Aquac..

[43] Springer A, Skrzypczak A (2015). The effect of above–water artificial light on the zooplankton abundance in cages for fish rearing. Pol. J. Natur. Sc..

[44] Loose CJ, Dawidowicz P (1994). Trade-offs in diel vertical migration by zooplankton: The costs of predator avoidance. Ecology..

[45] Gregory RS, Powles PM (1985). Chronology, distribution, and sizes of larval fish sampled by light traps in macrophytic Chemung Lake. Can. J. Zool..

[46] Fermin AC, Seronay GA (1997). Effects of different illumination levels on zooplankton abundance, feeding periodicity, growth and survival of the Asian sea bass, *Lates calcarifer* (Bloch), fry in illuminated floating nursery cages. Aquaculture.

[47] Rahel FJ, Nutzman JW (1994). Foraging in a lethal environment: Fish predation in hypoxic waters of a stratified lake. Ecology.

[48] Roberts JJ (2012). Evidence of hypoxic foraging forays by yellow perch (*Perca flavescens*) and potential consequences for prey consumption. Freshw. Biol..

[49] McKenzie EKG, Kwan GT, Tresguerres M, Matthews PGD (2022). A pH-powered mechanochemical engine regulates the buoyancy of *Chaoborus* midge larvae. Curr. Biol..

[50] Swift MC (1976). Energetics of vertical migration in *Chaoborus trivittatus* larvae. Ecology.

[51] Swift MC, Forwarder RB (1981). *Chaoborus* prey capture efficiency in the light and dark 1. Limnol. Oceanogr..

[52] Menzel, R. Spectral Sensitivity and Color Vision in Invertebrates In *Comparative Physiology and Evolution of Vision in Invertebrates. Handbook of Sensory Physiology*, Vol. 7 / 6 / 6 A (ed. Autrum, H. *et al.*) 503–580 (Springer, 1979).

[53] Levine JS, MacNichol EF (1982). Color vision in fishes. Sci. Am..

[54] Jachner A (1991). Food and habitat partitioning among juveniles of three fish species in the pelagial of a mesotrophic lake. Hydrobiologia.

[55] Fornshell JA, Tesei A (2013). The development of SONAR as a tool in marine biological research in the twentieth century. Int. J. Oceanogr..

[56] Garratt MJ, Jenkins SR, Davies TW (2019). Mapping the consequences of artificial light at night for intertidal ecosystems. Sci. Total Environ..

[57] Gaston KJ, Bennie J, Davies TW, Hopkins J (2013). The ecological impacts of nighttime light pollution: A mechanistic appraisal. Biol. Rev..

[58] Seymoure B, Dell A, Hölker F, Kalinkat G (2023). A framework for untangling the consequences of artificial light at night on species interactions. Philos. Trans. R. Soc. Lond. B Biol. Sci..

[59] Parma S (1969). Notes on the larval taxonomy, ecology, and distribution of the Dutch *Chaoborus* species (Diptera, Chaoboridae). Beaufortia.

[60] Nilssen JP (1974). On the ecology and distribution of the Norwegian larvae of *Chaoborus* (Diptera, Chaoboridae). Norsk ent. Tidsskr..

[61] Salmela J, Härmä O, Taylor DJ (2021). *Chaoborus flavicans* Meigen (Diptera, Chaoboridae) is a complex of lake and pond dwelling species: A revision. Zootaxa.

[62] R Core Team. R. A Language and Environment for Statistical Computing; R Foundation for Statistical Computing (Vienna, Austria, 2021).

[63] Bolker BM (2009). Generalized linear mixed models: A practical guide for ecology and evolution. Trends Ecol. Evol..

[64] Magnusson, A. *et al.* Package ‘glmmtmb’. R Package version 1.1.3 (2017). Accessed 14 March 2022.

[65] Fox, J. & Weisberg, S. *An {R} Companion to Applied Regression*, 2nd edn. Sage, Thousand Oaks CA (2011). http://socserv.socsci.mcmaster.ca/jfox/Books/Companion. Accessed 6 Nov 2021.

[66] Hartig, F. *DHARMa: Residual Diagnostics for Hierarchical (Multi-level/Mixed) Regression Models*. R package version 0.4.5 (2022). http://florianhartig.github.io/DHARMa/. Accessed 16 Jan 2022.

[67] Lenth, R., Singmann, H., Love, J., Buerkner, P. & Herve, M. 2020. Emmeans: Estimated marginal means, aka least-squares means. R package version **1**(1), 3. Available online: https://cran.r-project.org/web/packages/emmeans/emmeans. Accessed 4 Jan 2022.

[68] Therneau TM, Grambsch PM (2000). Modeling Survival Data: Extending the Cox Model.

[69] Therneau, T. M. A Package for Survival Analysis in R. R package version 3.5-3, https://CRAN.R-project.org/package=survival (2023).

[70] Bland JM, Altman DG (1998). Survival probabilities (the Kaplan–Meier method). BMJ.

[71] Cox DR (1972). Regression models and life-tables. J. R. Stat. Soc. Ser. B Stat. Methodol..

