# Supplementary material for: Artificial light at night bans Chaoborus from vital epilimnetic waters
Source: Sci Rep. 2024 Apr 5;14:7995. doi: 10.1038/s41598-024-58406-y (PMC10997633; doi:10.1038/s41598-024-58406-y)
Supplement: Supplementary file 1 — Supplementary Information. [file 41598_2024_58406_MOESM1_ESM.pdf]

Artificial light at night bans *Chaoborus* from vital epilimnetic waters

Mirosław Ślusarczyk, Anna Bednarska, Marcin Lukasz Zebrowski, Joanna Tałanda

Supplementary Material

Table 1. Planned contrast for estimated marginal means using a generalized linear mixed effects model to test the effect of depth range (0-5, 5-10, 10-15, 15-20, 20-25 m) on the abundance of *Chaoborus* larvae in the lake. Statistically significant differences (α = 0.05) are shown in bold (E - estimate, SE - standard error, df - degrees of freedom, T - T-test, p - p-value).

Contrast	E	SE	df	T	*p*
0-5m – 5-10m	-0.546	0.306	67	-1.785	0.239
0-5m – 10-15m	0.376	0.306	67	1.230	0.446
0-5m – 15-20m	**0.969**	**0.306**	**67**	**3.169**	**0.016**
0-5m – 20-25m	**0.993**	**0.306**	**67**	**3.245**	**0.015**
5-10m – 10-15m	**0.922**	**0.306**	**67**	**3.015**	**0.022**
5-10m – 15-20m	**1.515**	**0.306**	**67**	**4.954**	**<0.001**
5-10m – 20-25m	**1.539**	**0.306**	**67**	**5.031**	**<0.001**
10-15m – 15-20m	0.593	0.306	67	1.939	0.239
10-15m – 20-25m	0.617	0.306	67	2.016	0.239
15-20m – 20-25m	0.023	0.306	67	0.077	0.939

Table 2. Planned contrast for estimated marginal means using generalized linear mixed effects model to test the effect of depth (0-5, 5-10, 10-15, 15-20, 20-25 for each levels of treatment (Day – sunlight, Before – nighttime ahead, ALAN – ALAN, After – nighttime after) on the abundance of *Chaoborus* larvae in the lake Roś. Statistically significant differences (α = 0.05) are marked in bold (E – Estimate, SE – standard error, df – degrees of freedom, T – T-test, *p* – p – value).

Treatment	Contrast	E	SE	df	T	*p*
Day	0-5 m – 5-10 m	-0.447	0.708	67	-0.632	1.000
	0-5 m – 10-15 m	-0.690	0.708	67	-0.974	1.000
	0-5 m – 15-20 m	**-3.902**	**0.708**	**67**	**-5.512**	**<0.001**
	0-5 m – 20-25 m	**-4.974**	**0.708**	**67**	**-7.025**	**<0.001**
	5-10 m – 10-15 m	-0.243	0.708	67	-0.343	1.000
	5-10 m – 15-20 m	**-3.455**	**0.708**	**67**	**-4.880**	**<0.001**
	5-10 m – 20-25 m	**-4.527**	**0.708**	**67**	**-6.394**	**<0.001**
	10-15 m – 15-20 m	**-3.212**	**0.708**	**67**	**-4.537**	**<0.001**
	10-15 m – 20-25 m	**-4.284**	**0.708**	**67**	**-6.051**	**<0.001**

	15-20 m – 20-25 m	-1.072	0.708	67	-1.514	0.539
Before	0-5 m – 5-10 m	1.773	0.708	67	2.504	0.059
	0-5 m – 10-15 m	**4.233**	**0.708**	**67**	**5.979**	**<0.001**
	0-5 m – 15-20 m	**4.950**	**0.708**	**67**	**6.992**	**<0.001**
	0-5 m – 20-25 m	**5.233**	**0.708**	**67**	**7.391**	**<0.001**
	5-10 m – 10-15 m	**2.460**	**0.708**	**67**	**3.475**	**0.005**
	5-10 m – 15-20 m	**3.177**	**0.708**	**67**	**4.487**	**<0.001**
	5-10 m – 20-25 m	**3.460**	**0.708**	**67**	**4.887**	**<0.001**
	10-15 m – 15-20 m	0.717	0.708	67	1.013	0.630
	10-15 m – 20-25 m	1.000	0.708	67	1.412	0.487
	15-20 m – 20-25 m	0.283	0.708	67	0.399	0.691
ALAN	0-5 m – 5-10 m	-1.734	0.708	67	-2.450	0.085
	0-5 m – 10-15 m	-1.117	0.708	67	-1.578	0.358
	0-5 m – 15-20 m	1.615	0.708	67	2.280	0.103

	0-5 m – 20-25 m	**2.187**	**0.708**	**67**	**3.089**	**0.018**
	5-10 m – 10-15 m	0.617	0.708	67	0.872	0.773
	5-10 m – 15-20 m	**3.349**	**0.708**	**67**	**4.730**	**<0.001**
	5-10 m – 20-25 m	**3.922**	**0.708**	**67**	**5.539**	**<0.001**
	10-15 m – 15-20 m	**2.732**	**0.708**	**67**	**3.859**	**0.002**
	10-15 m – 20-25 m	**3.305**	**0.708**	**67**	**4.668**	**<0.001**
	15-20 m – 20-25 m	0.573	0.708	67	0.809	0.773
After	0-5 m – 5-10 m	-2.322	0.914	67	-2.540	0.067
	0-5 m – 10-15 m	-0.545	0.914	67	-0.596	1.000
	0-5 m – 15-20 m	2.184	0.914	67	2.390	0.079
	0-5 m – 20-25 m	**2.517**	**0.914**	**67**	**2.754**	**0.045**
	5-10 m – 10-15 m	1.777	0.914	67	1.944	0.168
	5-10 m – 15-20 m	**4.506**	**0.914**	**67**	**4.930**	**<0.001**
	5-10 m – 20-25 m	**4.839**	**0.914**	**67**	**5.294**	**<0.001**

10-15 m – 15-20 m	**2.729**	**0.914**	**67**	**2.986**	**0.028**
10-15 m – 20-25 m	**3.062**	**0.914**	**67**	**3.350**	**0.011**
15-20 m – 20-25 m	0.333	0.914	67	0.365	1.000

Table 3. Planned contrast for estimated marginal means using generalized linear mixed effects model to test the effect of treatment (Day - daytime, Before - nighttime before ALAN treatment, ALAN - artificial light at night, After - nighttime after ALAN treatment) for each depth range (0-5, 5-10, 10-15, 15-20, 20-25 m) on the abundance of *Chaoborus* larvae in the lake. Statistically significant differences (α = 0.05) are marked in bold (E – Estimate, SE – standard error, df – degrees of freedom, T – T-test, *p* – p – value).

**Depth**	**Contrast**	**E**	**SE**	**df**	**T**	***p***
0-5m	Day - Before	**-5.233**	**0.708**	**67**	**-7.391**	**<0.001**
5-10m		**-3.013**	**0.708**	**67**	**-4.255**	**<0.001**
10-15m		-0.310	0.708	67	-0.438	1.000
15-20m		**3.619**	**0.708**	**67**	**5.112**	**<0.001**
20-25m		**4.974**	**0.708**	**67**	**7.025**	**<0.001**
0-5m	Day - ALAN	**-2.387**	**0.708**	**67**	**-3.372**	**0.005**
5-10m		**-3.675**	**0.708**	**67**	**-5.190**	**<0.001**
10-15m		**-2.815**	**0.708**	**67**	**-3.976**	**0.001**
15-20m		**3.130**	**0.708**	**67**	**4.420**	**<0.001**
20-25m		**4.774**	**0.708**	**67**	**6.743**	**<0.001**

0-5m	Day - After	**-2.518**	**0.818**	**67**	**-3.079**	**0.006**
5-10m		**-4.392**	**0.818**	**67**	**-5.372**	**<0.001**
10-15m		**-2.373**	**0.818**	**67**	**-2.902**	**0.020**
15-20m		**3.569**	**0.818**	**67**	**4.366**	**<0.001**
20-25m		**4.974**	**0.818**	**67**	**6.084**	**<0.001**
0-5m	Before – ALAN	**2.846**	**0.708**	**67**	**4.019**	**0.001**
5-10m		-0.662	0.708	67	-0.935	0.707
10-15m		**-2.505**	**0.708**	**67**	**-3.538**	**0.004**
15-20m		-0.490	0.708	67	-0.692	1.000
20-25m		-0.200	0.708	67	-0.282	1.000
0-5m	Before – After	**2.716**	**0.818**	**67**	**3.322**	**0.005**
5-10m		-1.379	0.818	67	-1.687	0.289
10-15m		**-2.062**	**0.818**	**67**	**-2.523**	**0.042**
15-20m		-0.050	0.818	67	-0.062	1.000

20-25m		0.000	0.818	67	0.000	1.000
0-5m	ALAN – After	-0.130	0.818	67	-0.159	0.874
5-10m		-0.717	0.818	67	-0.877	0.707
10-15m		0.442	0.818	67	0.541	1.000
15-20m		0.439	0.818	67	0.537	1.000
20-25m		0.200	0.818	67	0.245	1.000

Table 4. Data on Sun and Moon Rise and Set Hours, Visible moon surface, and Cloud Cover during field tests.

Date	Sun set[1]	Sun rise[1]	Moon set[2]	Moon rise[2]	Visible moon surface[2]	Cloud cover[3]
4-5.7	21:04	4:10	22:31	7:10	2%	25%
15-16.7	20:55	4:23	19:59	3:41	96%	50%
16-17.7	20:54	4:24	20:48	4:38	99%	5%
22-23.7	20:46	4:33	11:18	23:15	78%	25%
23-24.7	20:45	4:34	12:27	23:31	70%	5%

Data source:

1- https://meteogram.pl/slonce/polska/pisz/

2 -https://meteogram.pl/ksiezyc/polska/pisz/

3- https://www.wolframalpha.com/

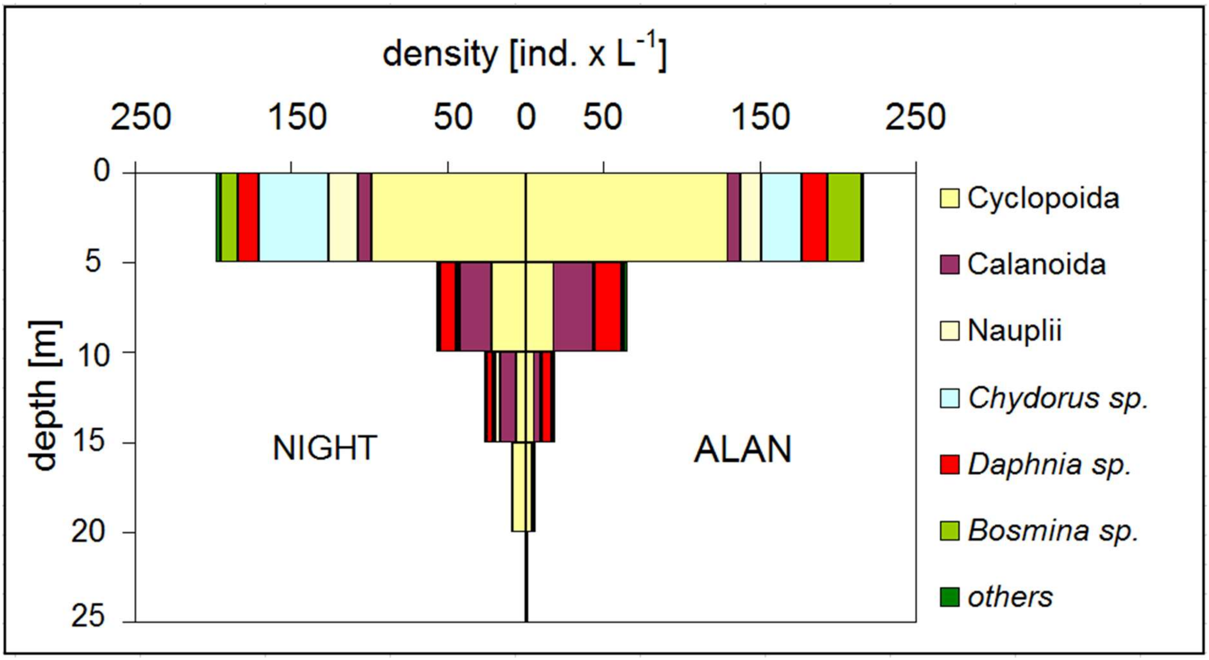

Figure 1. Example of vertical distribution of planktonic crustaceans - potential prey of *Chaoborus* larvae in the dark night before the experiment (left side) and during ALAN treatment (right side) in an experimental site in Lake Roś.

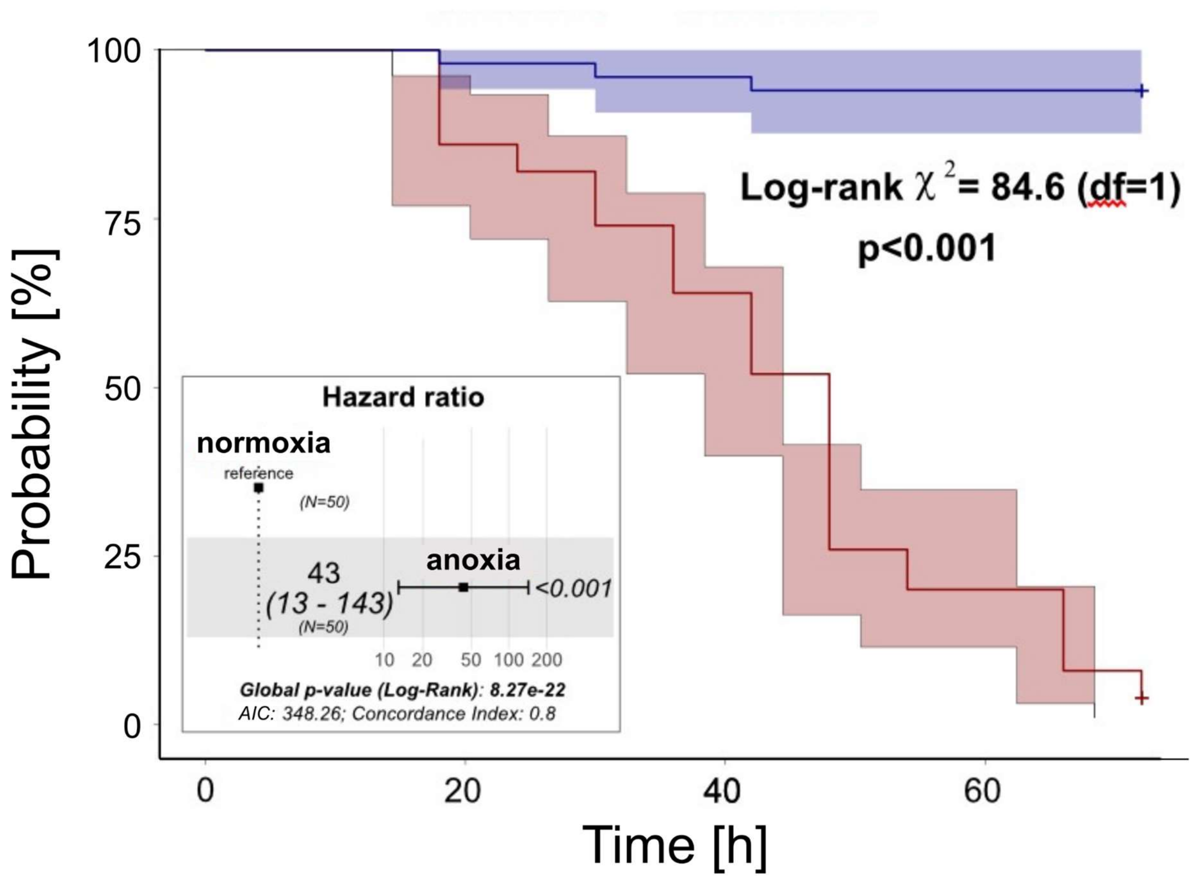

Figure 2. Kaplan-Meier estimators with 95% confidence bands for the survival of *Chaoborus* larvae under control and hypoxia conditions. The null hypothesis of no significant ($\alpha = 0.05$) difference in survival times between treatments was evaluated using the log-rank test. The effect size was assessed using the hazard ratio index and the Cox proportional hazards model (inset). The predictive power of the model is expressed by the concordance index (= 0.8; df - degrees of freedom. N=100).

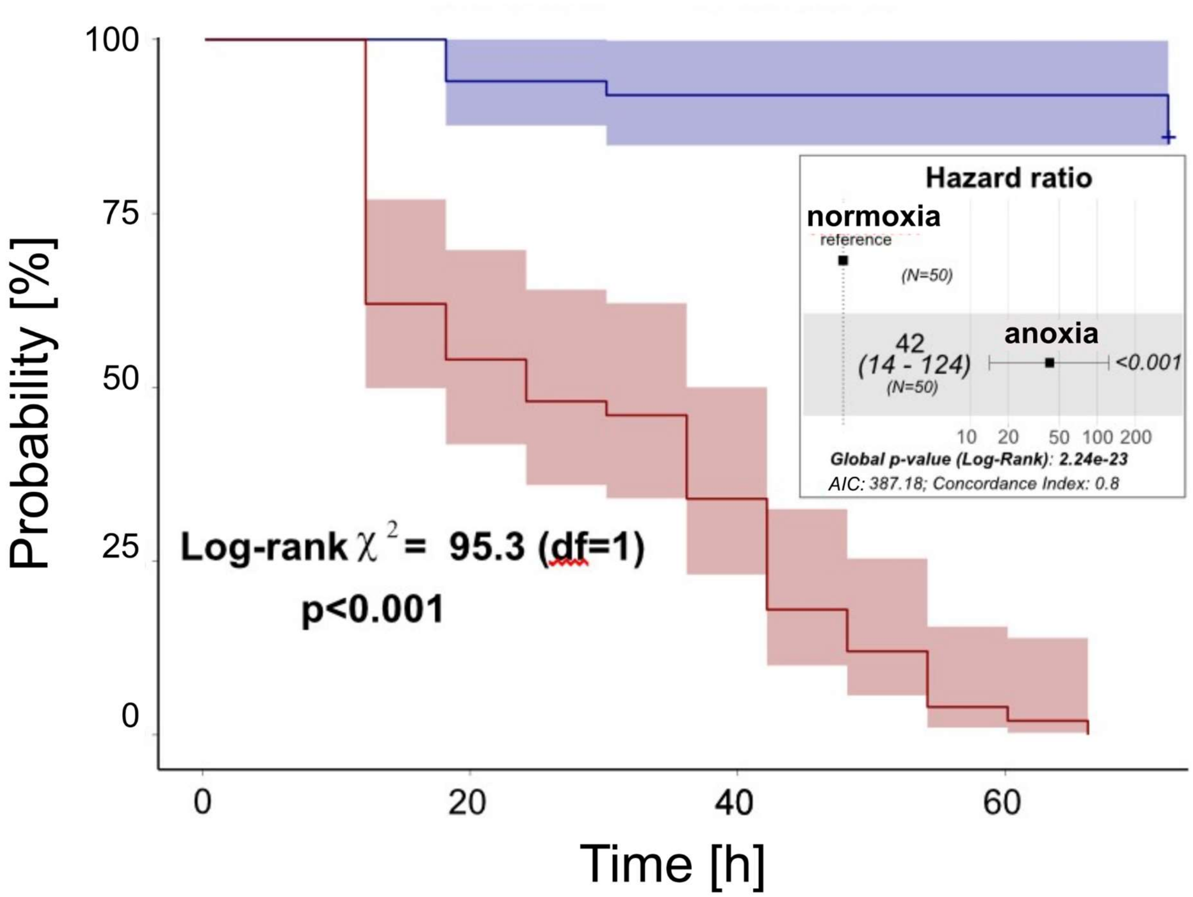

Figure 3. Kaplan-Meier estimators with 95% confidence bands for the persistence of Chaoborus larvae in the water column under control and hypoxia conditions. The null hypothesis of no significant ($\alpha = 0.5$) difference between the Kaplan-Meier curves for the treatments was tested using the log-rank test. The effect size was assessed using the hazard ratio index and the Cox proportional hazards model (inset). The predictive power of the model is expressed by the concordance index (= 0.8; df - degrees of freedom. N=100).

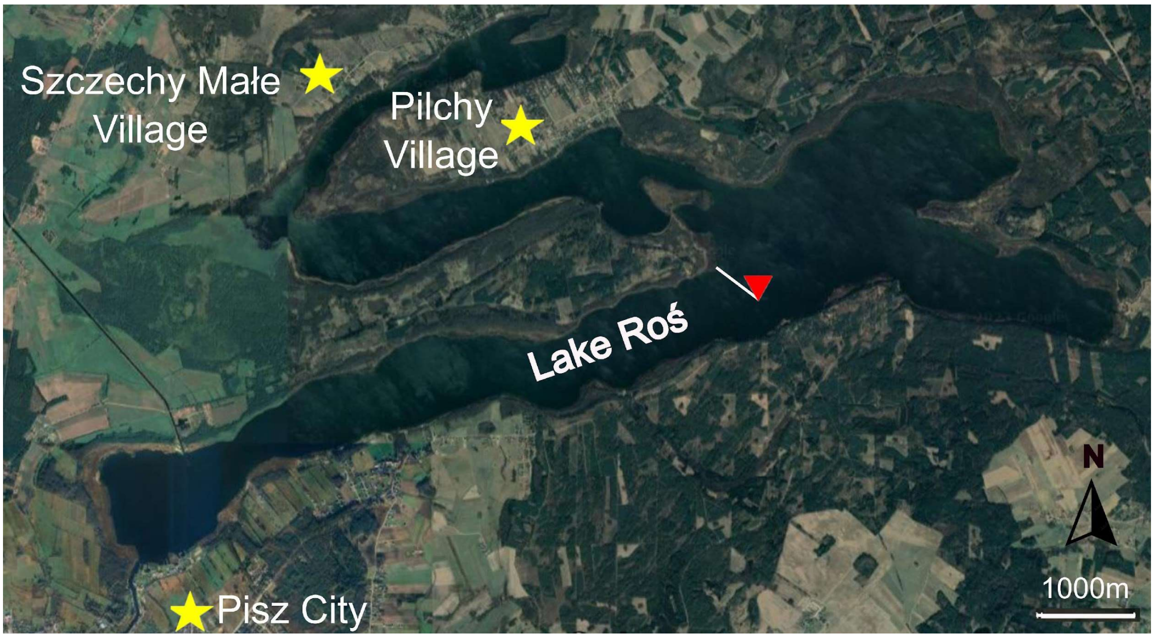

Figure 4. The map of the study area, Lake Roś. The red triangle indicates the position of the lamp during the experiment, the white line represents the transect (diurnal distribution of *Chaoborus* larvae in the lake from Fig. 2), yellow stars indicate more significant residential areas that emit artificial light at night. Map data ©2023 Google.

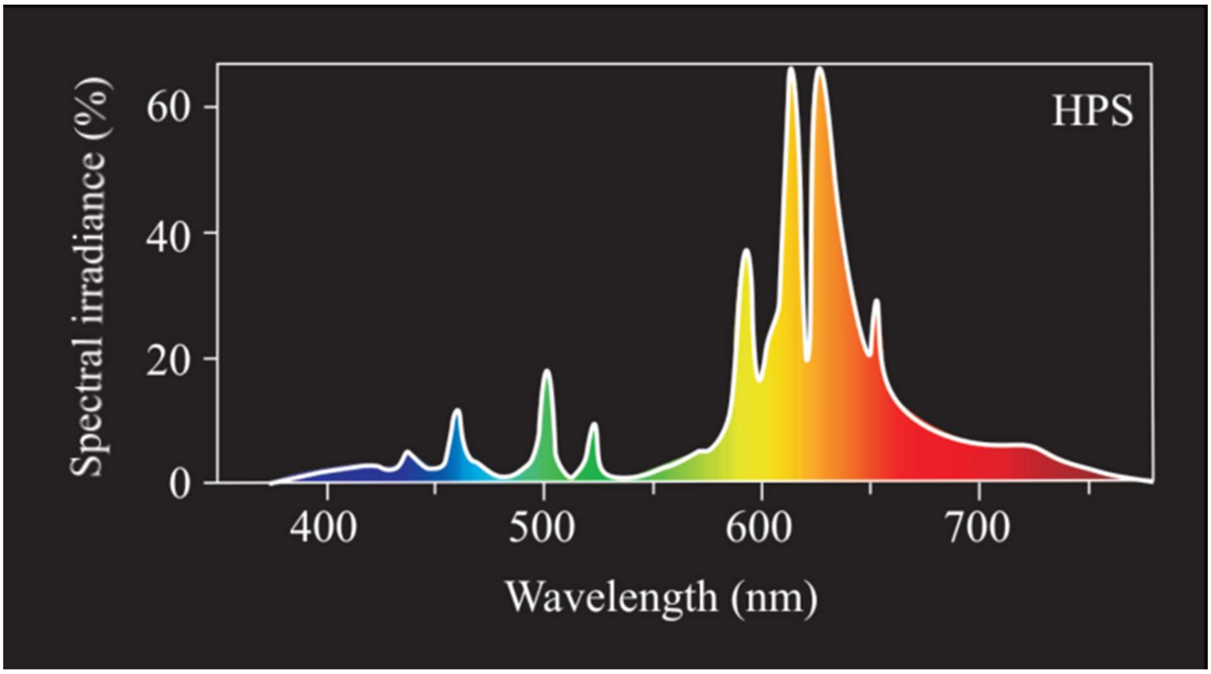

Figure 5. Spectral irradiance of the HPS lamp used as the artificial light source in the field test, measured with a UV-VIS spectrophotometer (USB 4000, Ocean Optics, Dunedin, Florida, USA).
